# The effects of socioeconomic conditions on old-age mortality within shared disability pathways

**DOI:** 10.1371/journal.pone.0238204

**Published:** 2020-09-03

**Authors:** Mathias Voigt, Antonio Abellán, Julio Pérez, Diego Ramiro

**Affiliations:** Center for Humanities and Social Sciences, Spanish National Research Council, Madrid, Spain; Universidade Federal de Minas Gerais, BRAZIL

## Abstract

**Objective:**

How disability manifests itself in an individual is a highly complex process influenced by a wide range of individual and environmental factors. Its complexity makes the search for generalizable characteristics of the disablement process a challenging task. Consequentially, little is known about how the effect on other health outcomes such as life expectancy are modified after the onset of chronic ailments. In this paper we posit an alternative approach to generalize health trajectories of older people with disability and then analyze how socioeconomic conditions affect the longevity within these trajectory groups.

**Methods:**

Individual level information about the first three successive onsets of chronic disability after age 50 is transformed into state-sequences. We extract trajectory groups based on onset time and the time spent in a certain state. Mortality hazards are then estimated with a Gompertz proportional hazards model to compare effects of different socioeconomic measures within the trajectory groups.

**Results:**

Three distinct trajectory groups are identified, the mild (1), the early severe (2), and late severe (3) pathway. Estimates of the mortality analysis suggest that social inequalities in longevity are less pronounced after onset of old-age disability. We found a consistent survival prolonging effect for individuals who engage in daily activities (such as meeting with friends, walking) that ranged between 33.2% and 77.3%. The importance of other variables varies between trajectory groups.

**Discussion:**

This study shows how health trajectories of individuals with disability can be generalized when information on the onset and severity of single conditions is available. Such an approach may help us to better predict health and care expenditures and help families and individuals with their personal care planning. The findings from the subsequent survival analysis suggest a substantial reduction of socioeconomic mortality differences after onset of old-age disability, which appears to be independent of its nature.

## Introduction

What happens after the onset of physical disability in mid or later life is often described as dynamic and sequential process that covers a variety of different aspects ranging from the underlying disease or conditions to environmental barriers [1]. Various individual and social factors are known to influence the pace and direction of trajectories between the onset of a chronic condition or physiological change and the potential functional consequences. This often age-associated process of loss of physical, cognitive, or sensorial functioning is modified by individual characteristics, such as education, cognitive status, or activity level and by the larger socio-cultural context [2,3].

Knowledge on generalizable characteristics and patterns of the disablement process can be important for the estimation of health and care resources as well as for assisting families and individuals with their personal care planning. While most trajectory-based approaches that have characterized disability as a progressive process have used growth models or self-selected pathway classifications [4–6], in this paper we explore an alternative approach to identify pathways of disability progression, which is based on the comparison of indifferences between individual age-sequenced health trajectories.

Our second objective is to use the knowledge about these pathways to analyze mortality differences within and between them in dependence of different social context variables. While the association between socioeconomic measures, late-life morbidity, or age-specific mortality is well-established, surprisingly little is known about how socioeconomic factors impact mortality and other health outcomes after onset of chronic disability and in the light of ongoing medical and social progress [7–9].

The linkage between survey data, directed at individuals with disabilities and their families, and register-based follow-up information on mortality allowed us to examine how these social conditions affect mortality risks after onset of later life disability and between different trajectory groups.

### Shared pathways

While most disability research is focused on the incidence/prevalence or transition rates, there has been an increasing number of studies addressing disability from a pathway perspective. The vast amount of health conditions, unobserved effects of multimorbidity, and different degrees of severity often hinder a uniform assessment of age-specific health status and make the classification of disability trajectories a challenging task [10,11]. To avoid these complications researchers have either relied on growth curve approaches or group based trajectory modeling [4,6,12,13].

Another way to classify disability pathways, is the retrospective assessment of trajectories preceding death [14,15]. The main advantage of such an approach, which uses information on extinct cohorts, is that estimates are not affected by a potential healthy survivor bias or informative censoring [16].

In this paper, we propose an alternative way to capture commonly shared patterns between individual level health trajectories. Therefore, we transform information about the onset and severity of a disability from a large, nationally representative health survey into state-sequence data and apply exploration tools used in sequence analysis to group individual trajectories by their relative indifference in terms of timing and duration in pre-defined health states.

### Inequalities in health and the onset of disability

Inequalities in later life health outcomes are partly derived from structural social differences, or in other words, processes of social stratification and its components such as social mobility and the changing distribution and value of resources. At an individual level there is strong evidence showing that the onset of chronic morbidity and levels of age-specific mortality are associated with the individual’s socioeconomic status, measured through income, occupation, education, and other socioeconomic dimension. Better educated and wealthier individuals generally experience a later onset of chronic, old-age morbidity and survive on average longer when compared with their less wealthy and lower educated [17–22]. It is assumed that such disparities ultimately derive from structurally restricted access to key determinants of health such as fair pay and quality education [23].

The mechanism which links these inequalities to health outcomes, such as the onset age of severe chronic conditions, is complex and not yet fully understood. Among the more popular hypotheses is the “cumulative disadvantage” hypothesis that posits that the latent, negative effects of sociocultural and economical disadvantages accumulate over the life course, lead to an increasing risk of being exposed to SES-associated risk factors such as smoking and lack of exercise, and manifest themselves in health inequalities later in life [24]. The pathways approach, on the other hand, explains this process as sequences of exposures to certain risk factors. The experience of certain unfavorable conditions is expected to increase the probability of being exposed to the next disadvantageous conditions in the following life sequence and ultimately determine the disease and mortality risk [25]. Other research highlights the importance of “critical” or “sensitive” periods, like the first years of life. Exposure to unfavorable social and health conditions during these periods is associated with permanent health disadvantages later on in life and have been linked to dramatically increased risks for serious health conditions, such as ischemic heart diseases, diabetes mellitus and mortality [26–28].

In this paper, our second objective is to examine how these social mechanisms determine health inequality after the onset of chronic morbidity and within previously defined disability pathways. Therefore, we analyze differences in age-specific mortality hazards by pathway group and different social context variables over a five-year period. The linked data sources we used for this analysis is described in the following section. Second, it is explained how the definition of disability is derived and how a matching algorithm can help to identify shared disability patterns based on the first three limitations onsets. Third, the statistical model for the survival analysis is explained. Estimated effects are then compared to mortality differences within the population without disability. Last, limitations and implications on future research are concluded and discussed.

## Methods and materials

### Data

The National Survey on Disability, Personal Autonomy, and Dependency (EDAD, Spanish: *Encuesta sobre*, *Discapacidad*, *Autonomía personal y Situaciones de Dependencia*), conducted by the Spanish National Institute of Statistics (INE) in 2008, is one of the largest national survey studies on the topics of health, disability, and care in Europe. Stratified samples for this cross-sectional study were drawn in a two-stage process, which led to a final sample size of 258,187 individuals (within 96,075 households) between the ages 0 and 104. Data was collected through face-to-face interviews between November 2007 and February 2008 (overall response rate 97%). When a household member aged 6 or older was identified as disabled, defined as substantive limitation in the performance of activities of daily life that last or are predicted to last for more than 1 year, the person or, if applicable, her caregiver was invited to answer an individual questionnaire directed at personal experiences with disability, which among others included questions about the exact age of onset of a disability as well as information on the levels of severity [29].

Building on the WHO International Classification of Functioning, Disability and Health (ICF), the questionnaires are directed at the efficiency of personal and technical support and the importance of social participation. Where possible, limitations are traced back to an underlying disease, chronic conditions, or injury [30,31].

The INE department for Socio-Demographic Statistics linked 207.529 individuals from the EDAD study to administratively collected mortality and exposure data. This longitudinal information was extracted from the annually updated statistics of natural population movements (MNP, Spanish: *Estadística de defunciones*. *Movimiento natural de la población)* and the national population register (Spanish: *Padrón*). The linkage allowed us to connect the retrospective information on health trajectories and disability status from the EDAD survey and discretely followed up mortality data for the period of five years immediately after the survey in 2008. For more information about the linkage see the (S1 Data).

We classified an individual “disabled” if he/she has experienced difficulties in performing at least one of 13 instrumental and basic activities of daily living [32,33]. Eight activities of daily living (ADL) are considered, which include moving inside, bathing, basic hygiene, urination (bladder control), toileting (bowel control), dressing, drinking, and eating. These are complemented by five instrumental activities of daily living (IADL), which include shopping, preparing meals, walking outdoors, using public transport, and being able to do housework.

### Characteristics of the study population

To observe only disability patterns representative of those commonly experienced in older individuals, the sample is restricted to all non-institutionalized individuals who experienced the onset of a disability after age 50. To prevent misclassifications when sequencing individual health trajectories, subjects are only included when their first onset occurred at least two years before the survey year (2008). The final sample contained 7335 cases (5071 females, 2264 males).

The average onset age of disability is 69 years for both men and women. The majority of men in both populations is married (76.3% in the disabled population; 83.3% in the population without disability). While most female participants in the disability-free population are married (67.7%), the majority of the women with disability is widowed (49.1% compared to 42.2% in marriage). 59.5% of the females and 48.8% of the males have no formal educational degree. Income, as additional measure of socioeconomic position is self-reported and measured as monthly gross income per household consumption unit (CU). Weights are derived from the household composition and assigned to every household member based on the OECD modified equivalence scale [34]. According to the distribution of monthly income per CU, 20–25% of the individuals with disability live from less than 500 Euro a month (females: 24.9%, males: 19.5%).

### Identifying shared pathways with sequence analysis and weighted clustering

To analyze dissimilarities between individual life courses after onset of disability and to identify underlying shared patterns that can then be examined and compared, different data mining techniques can be applied. Our approach is based on individual state sequences data that is grouped with minimal prior assumptions [35–37]. First, age-specific information on the number and severity of functional limitations is collected for the first three, successive episodes of disability onset after age 50. In other words, we order the retrospective information on the first three onsets after age 50, categorize the severity of each onset depending on the number of simultaneously occurred disabilities, and transform the data into a state-sequence format. Sensitivity checks confirmed that there was very little variation after the third onset, so we decided to only account for the first three events. The final data set contains age-specific information on transitions and trajectories based on the severity of disability at every single age up to the point, the individual died, the year 2006, or when the individual was right-censored (emigration).

**Fig 1:**
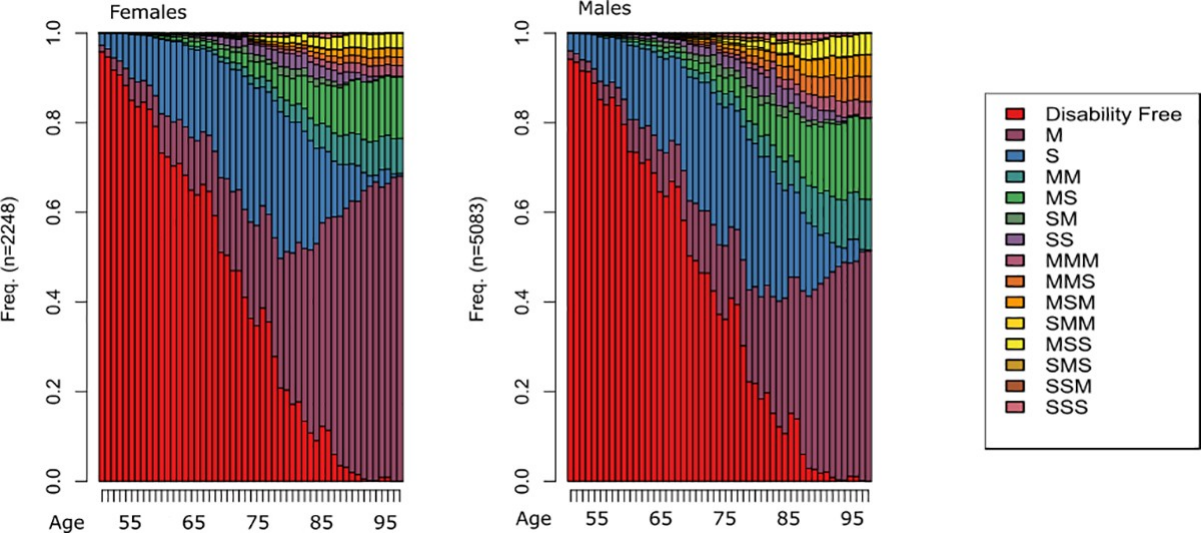
Sequence distribution by age and sex with legend.

Naturally, a majority of cases enters the study in the state “disability free” (DF) at age 50. Then over time, when individuals experience one or more onsets of disability, it will successively either leave them with a mild disability (M), defined as one additional limitation occurring after every new onset, or a severe disability (S) referring to the simultaneous onset of at least two different limitations or the onset of problems with food or drink intake. Fig 1 shows the relative distribution of possible states in the study population between the ages 50 and 100. These age-specific states range from one mild event (M) to three severe onsets (SSS).

Individual state sequences are formed under the assumption that an individual cannot recover from a disability. For example, an individual can never go back to the state “disability free” after the first onset of disability. We are aware that there is a chance that individuals with disabilities substantially benefit from rehabilitation programs and sometimes return to a baseline level of functionality [38]. Since we examine disability pathways in older individuals with ailments that have been prevalent for at least 12 months, the probability that individuals recover is very low [39].

A spell-length sensitive optimal matching algorithm (OM) is used to quantify levels of mismatch between each individual sequence of yearly updated disability information [40]. As measure of dissimilarities, this algorithm compares single trajectories with all other cases in a dissimilarity matrix and minimizes the residual variance between the state sequences [41,42]. The OM distances proposed by this measure are sensitive to differences in duration spent in a particular state. This has the advantage that it better distinguishes between long-term continuity and rapid changes of states [43]. Based on the dissimilarity matrix, several potential clustering options can be extracted. They are assessed and compared with the Average Silhouette Width (ASW), which is a within-group coherence measure that accounts for sequence dissimilarities within each group [42]. The analysis of dissimilarities and the clustering was conducted using the tools provided in the *TraMineR* and *WeightedCluster* R-packages [44,45].

### Regression model

Mortality hazards by single age are then estimated for a five-year period using a Gompertz proportional hazards survival model (*h*(*t*) = *a* * exp^(*bx*)^). The Gompertz model assumes that the logarithm of mortality risks grows linearly with increasing age. It has been shown repeatedly that it models human adult mortality accurately with only two unknown parameters [46]. The models are stratified by previously identified disability trajectory and sex. Regression coefficients, which are estimated through Maximum Likelihood Estimation (MLE), are asymptotically normal distributed. When comparing mortality between different subpopulations, proportional hazards approaches do not allow for capturing that variances in life lengths are inversely related to average life expectancy. In other words, the proportional hazards assumption imposes the same variance in length of life on all observed subgroups, which is often the opposite to what is observed in reality [47]. While this behavior often requires to adapt the model with an additional frailty term, only one model (disability-free population) was found to be affected. As the proportional hazards assumption was found to hold for the large majority of variables in all models, it was refrained to include an additional individual frailty term into the models [48].

As individuals enter the study at different ages, the time under risk of dying before the start year of the study remains unobserved and it is therefore necessary to account for left truncation [49]. In other words, individuals are selected based on their survival upon two years before the EDAD survey. To reduce the bias due to unobserved timing effects, all models are controlled for birth cohort.

## Results

**Fig 2:**
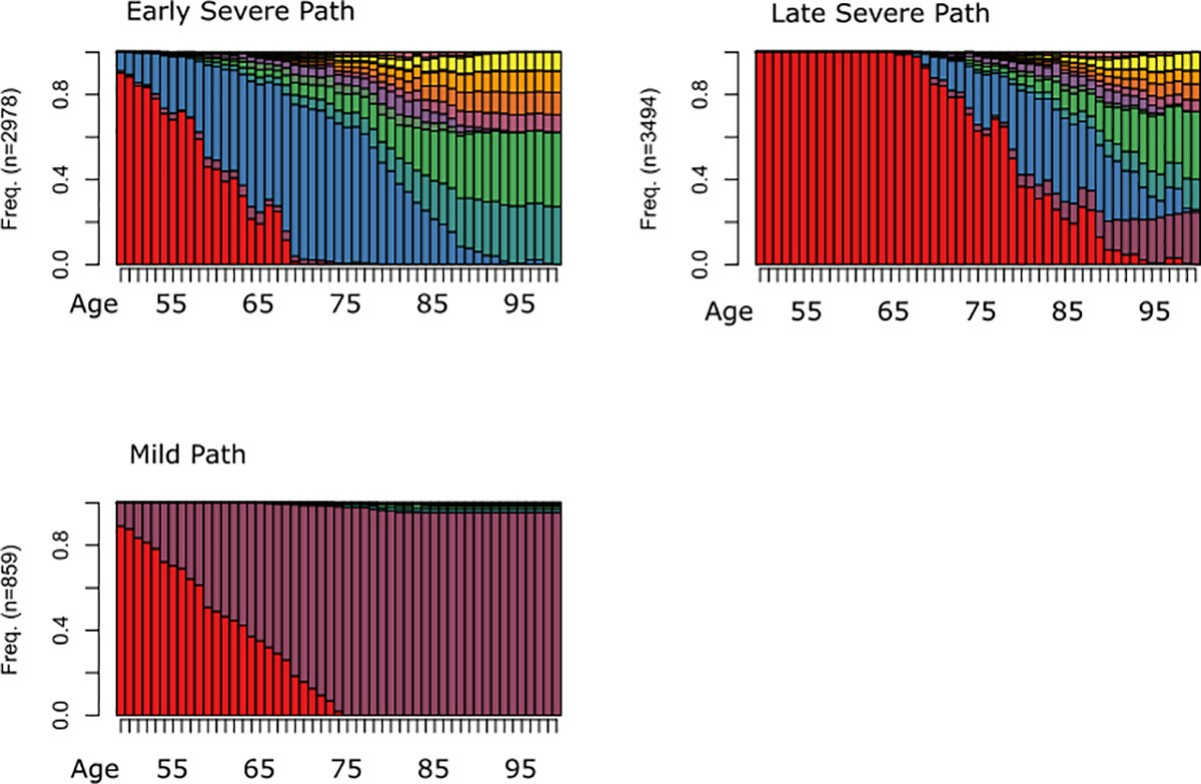
Relative distribution of state sequences by pathway and age.

The results from the ASW suggest that three clusters are optimal to represent the variation in individual disability trajectories in the data. These three pathways are labeled after a visual assessment of the relative state distributions at different ages. Generally, we observe the same three pathway groups for both sexes, but females experience a slightly earlier onset of disability. The relative distribution of states by pathway and age are shown in Fig 2.

**Fig 3:**
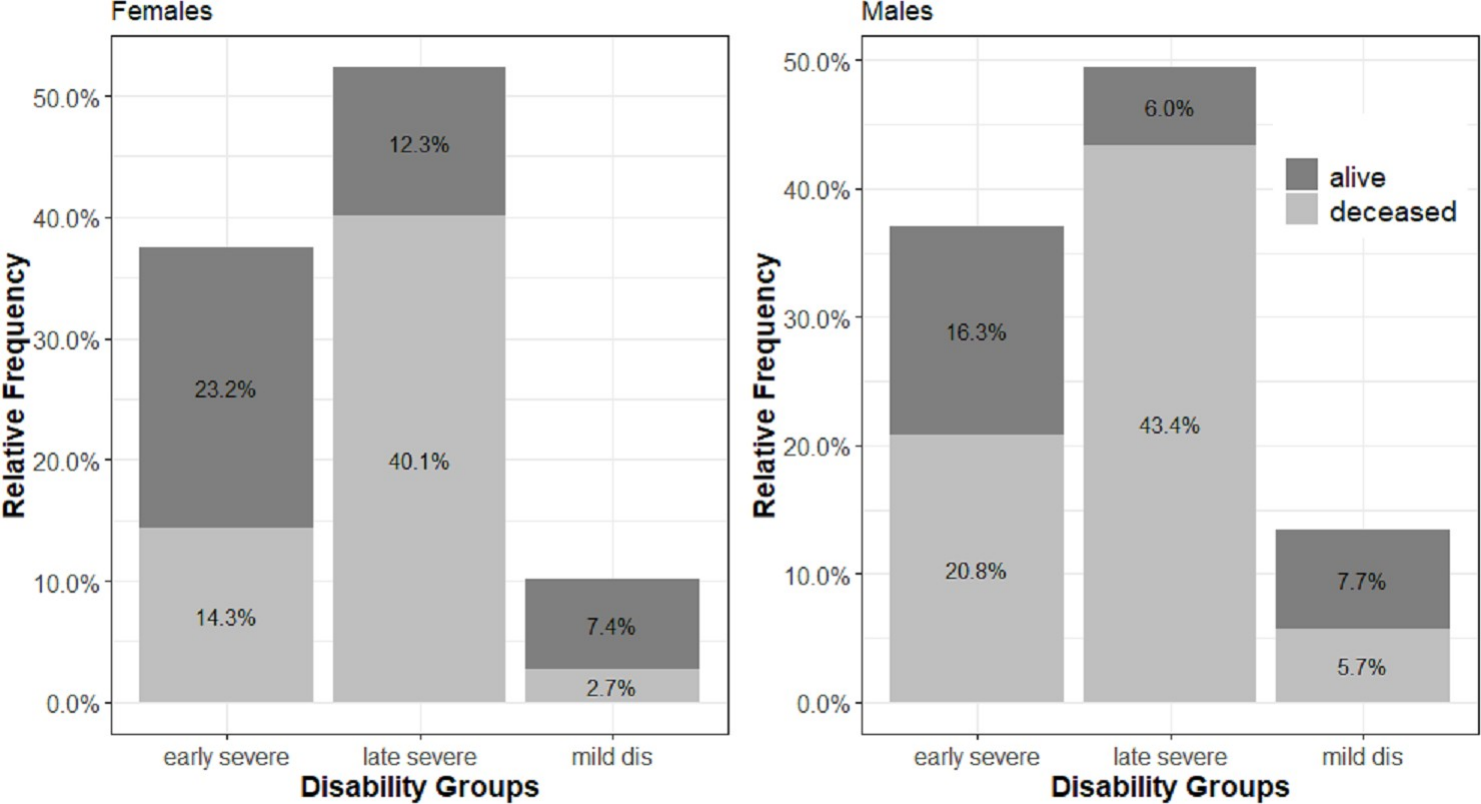
Relative distribution of the event of interest depending on the assigned disability trajectory.

The relatively small mild disability group consist of individuals with one mild onset (defined as 1 new disability at time of the event) and who did not have experienced a second event within a period of at least two years. The exception is a small proportion of individuals who experienced further episodes of disability at higher ages. Individuals in the other two groups have, on contrary, either experienced a severe onset of disability, defined as 2 or more limitations occurring at the same age, or have suffered from multiple successive events (disability onsets). These two groups can generally be distinguished by the timing of first onset and are therefore labeled early severe and late severe. Although the relative distribution plots suggest that individuals in these two groups experience multiple onsets of disability at higher ages, on average, they spent most of their time under observation in the state S, which indicates a single, severe onset. In fact, only 40% of the individuals in the early severe group have experienced a second event within the observation period. Within the late severe group, men appear to be less likely (51%) to experience from a second onset than women (60%). In Fig 3 the relative distribution of individuals is presented by sex and pathway group.

The colors can further help to make a first descriptive assessment of mortality by group and sex; the darker shade represent the proportion of survivors at the end of the follow-up period and the lighter shades those who have died. The graphs suggest that the late severe group experiences the highest fatality rates in the follow-up time while most individuals assigned to the mild trajectory survive until the end of the observation period. Across all trajectory groups, females exhibit a higher probability of surviving to the end of the study period compared to their male counterparts.

When comparing the mortality between pathway groups, differences in the age distribution within each group needs to be accounted for. The late severe group is with a mean age of 84 years substantially older than the two others (71 years for mild disability, 72 years for early severe), which indicates a higher risk of selection upon survival. The relative number of deaths by pathway group follow the same gradient and support the assumption. This issue is accounted for by comparing age-specific survival, within pathway groups.

### Mortality differences within pathway groups

Due to substantial differences in age distribution, individuals who fall into the same trajectory group should only be compared to other individuals in that same group. While comparability between groups is intended, in spite of using the same set of covariates, the comparison of effect sizes between disability trajectories might not be meaningful due to different underlying baseline hazard distributions. Four sets of nested Gompertz PH regression models are presented in tables below. These models are stratified by disability pathway and sex. Tables 1, 2 and 3 contain estimated hazard ratios, 95% confidence intervals, and overall model statistics for a survival analysis of individuals who are selected into the *early severe*, *late severe*, and *mild* disability pathway group.

**Table 1:** Gompertz PH regression models estimates–individuals with an early severe disability trajectory (stratified by sex).

	Dependent variable: Relative risk of dying		
	(1)	(2)	(3)
No daily activity	1.332***	1.326***	1.511***
*Reference*: *Daily Active*	(1.137, 1.561)	(1.132, 1.554)	(1.278, 1.788)
Suffers from multiple diseases	1.335*	1.225	1.019
*Reference*: *No multimorbidity*	(1.052, 1.695)	(0.973, 1.543)	(0.817, 1.272)
Birth Cohort	0.920***	0.920***	0.929***
	(0.898,0.943)	(0.898, 0.943)	(0.905, 0.953)
Income per CU *<* 500 Euro		0.926	0.820+
		(0.751, 1.141)	(0.649, 0.999)
Income per CU 500–750 Euro		0.987	0.969
*Reference*: *>750 Euro*		(0.831, 1.172)	(0.806, 1.164)
Incomplete Educ.		1.238	1.379*
		(0.970 1.579)	(1.074, 1.771)
Primary Educ.		1.348*	1.140
*Reference*: *Secondary/Higher Educ*.		(1.048, 1.734)	(0.873, 1.487)
Widowed			1.379**
			(1.125, 1.691)
Div./Single *Reference*: *Married*			1.346*(1.019, 1.776)
No close relatives			1.304
			(0.751, 2.264)
Close kin in same HH			1.180
			(0.896, 1.553)
Close kin in same municipality			1.209
*Reference*: *Kin in other geogr*. *area*			(0.924, 1.583)
Observations (Events)	2072 (690)	2072 (690)	2072 (690)
Likelihood Ratio Test (df total/additional)	147.335*** (6/3)	-4.190 (11/4)	20.913*** (15/5)
AIC	5435.94	5448.13	5437.22

+p<0.1

*p<0.05

**p<0.01

***p<0.001

**Table 2:** Gompertz PH regression models estimates–individuals with a late severe disability trajectory (stratified by sex).

	Dependent variable: Relative risk of dying		
	(1)	(2)	(3)
No daily activity	1.773***	1.773***	1.768***
*Reference*: *Daily Active*	(1.589, 1.979)	(1.588, 1.980)	(1.583, 1.975)
Suffers from multiple diseases	1.224**	1.221**	1.227**
*Reference*: *No Co-morbidity*	(1.068, 1.403)	(1.065, 1.399)	(1.069, 1.407)
Birth Cohort	0.971***	0.968***	0.973***
	(0.956, 0.987)	(0.953, 0.984)	(0.958, 0.990)
Income per CU *<* 500 Euro		0.977	0.981
		(0.866, 1.102)	(0.870, 1.107)
Income per CU 500–750 Euro		1.010	1.040
*Reference*: *>750 Euro*		(0.908, 1.123)	(0.933, 1.159)
Incomplete Educ.		0.919	0.914
		(0.782, 1.079)	(0.777, 1.074)
Primary Educ.		0.913	0.915
*Reference*: *Secondary/Higher Educ*.		(0.768, 1.084)	(0.770, 1.087)
Widowed			1.083
			(0.964, 1.217)
Div./Single			1.179
*Reference*: *Married*			(0.959, 1.448)
No Close Relatives			1.361*
			(1.041, 1.780)
Close kin in same HH			1.305**
			(1.094, 1.556)
Close kin in same municipality			1.229*
*Reference*: *Kin in other geogr*. *area*			(1.281, 1.468)
Observations (Events)	2530 (1884)	2530 (1884)	2530 (1884)
Likelihood Ratio Test (df total/additional)	157.021*** (6/3)	1.535 (12/6)	14.617*(16/4)
AIC	10720.95	10727.42	10722.8

+p<0.1

*p<0.05

**p<0.01

***p<0.001

**Table 3:** Gompertz PH regression models estimates–individuals with a mild disability trajectory (stratified by sex).

	Dependent variable: Relative risk of dying		
	(1)	(2)	(3)
No daily activity	1.654*	1.442+	1.431
*Reference*: *Daily Active*	(1.159, 2.359)	(1.001, 2.078)	(0.994, 2.059)
Suffers from multiple diseases	1.532	1.595	1.588
*Reference*: *No Co-morbidity*	(0.896, 2.359)	(0.909, 2.798)	(0.975, 2.780)
Birth Cohort	0.898***	0.904***	0.910***
	(0.847, 0.953)	(0.8582, 0.959)	(0.858, 0.964)
Income per CU *<* 500 Euro		1.841*	1.885*
		(1.152, 2.944)	(1.162, 3.059)
Income per CU 500–750 Euro		0.904*	0.993
*Reference*: *>750 Euro*		(0.852, 0.959)	(0.614, 1.604)
Incomplete Educ.		0.783	0.763
		(0.474, 1.291)	(0.464, 1.253)
Primary Educ.		0.547+	0.509*
*Reference*: *Secondary/Higher Educ*.		(0.312, 0.959)	(0.292, 0.885)
Widowed			1.021
			(0.632, 1.649)
Div./Single			1.366
*Reference*: *Married*			(0.746, 2.502)
No Close Relatives			1.229
			(0.456, 3.307)
Close kin in same HH			1.000
			(0.577, 1.733)
Close kin in same municipality			0.786
*Reference*: *Kin in other geogr*. *area*			(0.463, 1.335)
Observations (Events)	589 (125)	589 (125)	589 (125)
Likelihood Ratio Test (df total/additional)	31.693*** (6/3)	13.951+ (12/6)	3.399 (15/3)
AIC	1112.867	1106.916	1113.517

+p<0.1

*p<0.05

**p<0.01

***p<0.001

All presented models are significantly different from a model without additional covariates. Goodness of fit tests suggest that additional socioeconomic and household composition variables do not improve the model fit. The most robust effects over all models are the impact of birth cohort and daily activity. For individuals in the *early severe* trajectory estimates (Table 1) suggest that mortality hazards for those who are not daily active are between 32.2 and 51.1 percentage point higher when compared to those who are inactive. While mortality in the *early severe* group does not appear to be affected by multimorbidity, being born in an older cohort is suggested to have an increasing effect on the hazard in every pathway group. The effects of educational attainment and income remain inconclusive for the *early severe* pathway. Estimates in the most complete model (3) suggest an about 30 percentage point increased relative risk of dying for those in lowest income category when compared to the ones with the highest income. The effects for the household composition and civil status, added in the last model, indicate a protective effect of marriage for this group (37.9 percentage points higher relative risks for windows and a 34.6 percentage point higher relative risks for singles and divorced when compared to married individuals).

The model results for the *late severe* group, represented in Table 2, show slightly different importance in terms of the impact of single impact factors. Estimates suggest a strong, negative effect of lack of daily activity. Effect sizes vary between 76.8 and 77.3 percentage point higher risks between the different models. In contrast to the *early severe* group, suffering from additional diseases and chronic conditions is found to increase mortality hazards by about 22 percentage points in all models, suggesting that an aging related processes is at work that increases the risk of multimorbidity and its consequences. A further difference can be found in the effect of the proximity of a close kin network. The estimates suggest an increased hazard for both those without any close relatives and those with relatives living very close (or in the same household) when compared with individuals whose relatives live in a different municipality.

The Gompertz models results for *mild disability* trajectory group suggest that besides the birth cohort effects none of the included variables does significantly affect the mortality hazards. This is possibly related to the relatively small case numbers in this group. Among all factors, falling into the lowest income category is suggested to increase the hazard of dying by 84 to 88 percentage points.

To confirm the relative importance of socioeconomic measures on mortality at older ages, we estimated further models where we included individuals aged 50 and older who were disability free. As the survey was directed at the people with disability, we had only a reduced set of variables. The results, which are displayed in S2 Table, confirm often reported negative effects of low income and incomplete education. The estimates for the monthly income per CU show a between 8.6 and 22.5 percentage point increased hazard for survival in the lowest income category. There is also a strong, significant effect of low levels of education. Those without completed education are found to experience an 8.8 to 24.4 percentage point higher risk of dying within the follow-up period when compared to those with secondary or higher education. The results further suggest a higher risk of widowed and divorced or single individuals when compared with married ones. The estimated hazards are 42.3 percentage points higher for widows and 63.7 for divorced and singles respectively. Furthermore, estimates suggest an 8.5 percentage points higher hazard for those who live in a household with more than one other person.

## Discussion

Although the disablement process is influenced by a wide range of individual characteristics and constantly affected by macro-level changes such as medical breakthroughs or sociopolitical measures, knowledge on how this process can be generalized and if individuals can be placed in defined trajectory groups will lead to a better understanding of the probabilities and potential scenarios after the onset of chronic morbidity. Such knowledge can be used to quantify health equity issues, prepare public health interventions and better predict future health care costs.

In an attempt to provide a replicable methodology for identifying shared disability trajectories, our work describes how state sequence data based on the numbers of limitations and onset times of disability events can be used to feed an adapted optimal matching algorithm. This algorithm then compared individual age-sequenced disability experiences by onset age and length of state durations to identify potential pathways. Assessment tools like the ASW allow us to reduce the number of possible outcomes with some degree of confidence.

In our study, we find three distinct pathways that describe the majority of the observed patterns after the onset of a chronic disability. The smallest group in both sexes is the *mild* pathway, to which individuals got assigned to when they experienced the onset of one mild limitation and remained in this state for most of the follow up period. Individuals who were placed in the other two pathways often have suffered from multiple onsets or at least one severe onset. The *early severe* and the *late severe* trajectory can be distinguished by the average onset age of the first chronic disability.

Although limited to a small set of variables, the results from the subsequent analysis of mortality disparities suggest that 5-year survival in each pathway group is determined by different impact factors. Apart from the cohort effect, only a low household income was found to increase the hazard of dying in the *mild* pathway. The most robust finding for the other two pathway groups, besides that older cohorts are exposed to higher age-specific mortality rates, is the positive effect of daily activity on survival. Inactive individuals experienced an up to 77 percentage point higher relative risk compared to those who were conducting day-to-day tasks. Although sedentary lifestyles and the lack of daily activity have been repeatedly linked to early onset of disability, the importance of this finding should not be understated [50–52]. These results suggest an underlying health-promoting effect of community and active social network. While the ability to perform daily activities is a condition for maintaining friendships and other personal relationships, it has frequently been shown that an active social network gives incentives to be active yourself, which is linked to an improved health in older adults [53,54].

While our findings do not unambiguously confirm the aforementioned accumulated disadvantage or pathway hypotheses, the higher relative mortality risks for inactive individuals in the *late severe* path suggest that opportunity costs for adaptation to a life with disability might increase in older ages. Compared to those in the *early severe* group, who might be able to lean on larger networks and have found meaning in alternative activities that keep themselves occupied and active within their social networks, such an adaptation process appears to be more difficult when limitations occur later in life. As the decline of physical activity generally accelerates with older ages, it will be important to identify these high-risk groups and find ways to postpone or slow this process down [55]. While more accessible build-in environment are found to peak daily activity in older adults, small incentives such as diaries and digital step counter are found to efficiently boost the levels of daily activity often even independent of the kind of functional limitations [56–58].

Once individuals have experienced the onset of disability, our results suggest that the impact of social position on their survival is muted and that early or mid-life conditions might play less of a role. At the same time, we observed well-known mitigating effects of high income and higher education in household members without disability (S1 Table—Gompertz PH Regression Models Estimates–Individuals Free of Disability (stratified by sex)). Such a finding might be highly relevant for the research on health inequalities. While the occurrence of chronic disability was found to be highly dependent on the individuals’ socioeconomic position [22,59], in our study, disability appears to make individuals more equal for the time after the first onset. To assure that the small socioeconomic differences in mortality after onset of chronic disability are not entirely caused by health selection mechanisms, more research will need to further disentangle the influence of socioeconomic context on disability and survival.

There are several limitations due to the structure of the data and methodological choices. As there is just one cross-sectional time point from which information on disability and socioeconomic measures is obtained, changes in the health status during the follow-up period remain unobserved. Such changes would presumably have affected the disability free or *mild* category who might have experienced further events that would have placed them in one of the other categories. The disability-free population might not resemble the total population and age-specific mortality rates might differ due unobserved changes and selectivity as individuals living in a household with a dependent or disabled relative are exposed to higher levels of stress, negatively affecting their health and ultimately their risk of dying [60,61].

Moreover, there are numerous algorithms and methods that allow for grouping age-sequenced trajectories. Some of these methods do not produce reliable results or place the same individuals in different trajectory groups when the process is repeated [62]. Algorithms for comparing state sequences are always based on a simplification of the data, which might influence the assignation of individuals to one group or another. Therefore, selecting the right method might not always be straightforward and depends on the focus of the analysis. As sequences may be more similar regarding one aspect, such as duration, they may differ more when focusing on another [43]. With the focus on dissimilarities by the time spent in a distinct successive disability state, it can for example not be excluded that small differences in the selected population would have led to different assignation of individuals to the *early* or *late severe* group, especially considering that the onset age was close to the threshold age where these groups are separated.

The validity of the results might further be affected by the aforementioned selection mechanism. It is possible that selection upon survival has biased the risk to be part of the study population and the different pathway groups. It can further be assumed that when information on dates is gathered in face-to-face interviews with either very old people or their representatives, there will be a certain recall bias and possibly some degree of digit preference with respect to onset dates [63]. As the age at onset of disability was derived from this information it might be affected by these biases. The representativeness our results might be further limited for individuals at very high ages due to the exclusion of individuals who were registered in institutions like nursing homes. While the percentage of institutionalized individuals aged 65 and older in Spain is with about 3% relatively small, this percentage increases to about 17% for those above age 90. Although still a minority the institutionalized population might represent a much larger share of the total disabled population [64]

Nevertheless, the results confirm that there are underlying shared mechanisms that allow for grouping disability trajectories that then can be used to analyze the implications of a certain disability pathway on various outcomes such as the comparison of survival probabilities by different impact factors.

This research needs to be expanded on to answer questions like what determines the ability to adapt to certain disabilities and how does mental health relate to these processes [65]. The assessment of different trajectories across different health states or markers for different pathways through disability will remain important. From an economical perspective, it might help to better predict future care costs [66]. If, for example, the distribution of individuals in different pathways would change from time *a* to time *b*, it will affect average health spans. If there were for example relatively more individuals with a *late severe* pattern, this would change many factors including the demand of care just due to their higher mortality compared to the other pathway groups. At an individual level further analyses like this might help to facilitate decisions regarding care or more general about how to manage different domains of life after onset of disability [67–69].

## Supporting information

S1 Table Gompertz PH regression models estimates–individuals free of disability (stratified by sex). (DOCX)

S2 Table Gompertz PH regression models estimates–individuals free of disability (stratified by sex). (DOCX)

S1 Data On data linkage. (DOCX)
