# Supplementary material for: The effects of socioeconomic conditions on old-age mortality within shared disability pathways
Source: PLoS One. 2020 Sep 3;15(9):e0238204. doi: 10.1371/journal.pone.0238204 (PMC7470411; doi:10.1371/journal.pone.0238204)
Supplement: S1 Table — (DOCX) [file pone.0238204.s001.docx]

# S1 Table - Gompertz PH Regression Models Estimates – Individuals Free of Disability (stratified by sex)

|  | Dependent variable: Relative risk of dying | |
| --- | --- | --- |
|  | (1) | (2) |
| Birth Cohort | 0.895*** | 0.906*** |
|  | (0.886, 0.904) | (0.898, 0.914) |
| Health Limitations  *Reference: No Health Problem* | 1.763*** (1.622, 1.916) | 1.676 *** (1.557, 1.804) |
| Income per CU *<* 500 Euro | 1.334*** | 1.185** |
|  | (1.225, 1.457) | (1.095, 1.804) |
| Income per CU 500-750 Euro | 1.056 | 1.157*** |
| *Reference: >750 Euro* | (0.981, 1.135) | (1.085, 1.235) |
| Incomplete Educ. | 1.190*** | 1.173** |
|  | (1.086, 1.304) | (1.085, 1.269) |
| Primary Educ. | 1.044+ | 1.037+ |
| *Reference: Secondary/Higher Educ.* | (0.954, 1.143) | (0.959, 1.120) |
| Widowed |  | 1.315***  (1.217, 1.422) |
| Div./Single  *Reference: Married* |  | 1.610***  (1.479, 1.751) |
| >2 people in HH |  | 1.048+ |
| *Reference: 1-2 people in HH* |  | (0.988, 1.115) |
| Observations | 51,678 | 51,678 |
| LR Test (df total/additional) | 5834.55*** (9/9) | - 528.77 (12/3)*** |
| AIC | 51370.29 | 51370.29 |
| Note: +p<0.1; ∗p<0.05; ∗∗p<0.01; ∗∗∗p<0.001 | | |
