# Supplementary material for: The effects of socioeconomic conditions on old-age mortality within shared disability pathways
Source: PLoS One. 2020 Sep 3;15(9):e0238204. doi: 10.1371/journal.pone.0238204 (PMC7470411; doi:10.1371/journal.pone.0238204)
Supplement: S1 Data — (DOCX) [file pone.0238204.s003.docx]

# S1 – On Data Linkage

The dataset presented here was produced in answer to an agreement between the INE department for sociodemographic statistics and the IEGD (CCHS-CSIC) in 2017. Members of different INE departments linked three data sources to obtain the above-presented longitudinal data base. A personal identifier, such as the national ID number (DNI/NIE), was used in an attempt to link the 258,187 individuals who participated in National Survey on Disability, Personal Autonomy, and Dependency (EDAD, Spanish: *Encuesta sobre Discapacidad, Autonomía personal y Situaciones de Dependencia*) with the monthly updated Spanish population register (Spanish: *Padrón*) and the statistics for natural population movement (MNP, Spanish: *Movimiento natural de la población*). The information from the *Padrón* was used to assess the timing of changes in the household sizes. The MNP, which combines individual level information from various civil registers, contains more detailed information on the causes for the increase or reduction of household size. In the case of a reduction of household size the MNP data allowed for distinguishing if such a reduction was caused by the emigration or death of a household member.

As the EDAD survey was not designed to be linked to other data sources or registers, a substantial number of individuals could not be identified due to missing id information. As INE informed us, 50,658 individuals who had had participated in the survey had no personal identifier. It is assumed that this information is missing at random. 1783 individuals with valid id information in the EDAD could not be unequivocally matched with individuals in the registers and were excluded from the analysis. 1744 of these cases could not be linked to the MNP but have “left” the population according to the *Padrón* information. Many of these cases are presumably unregistered deaths, which might not yet have been recorded in the MNP.

More information about the sampling and underlying methodology of the EDAD survey can be found on the INE website (information in Spanish; Link: [Metodología EDAD.](https://www.ine.es/metodologia/t15/t1530418.pdf) The INE website further contains additional information about the *Padron´* [(Information](http://www.ine.es/dyngs/INEbase/es/operacion.htm?c=Estadistica_C&cid=1254736177011&menu=resultados&idp=1254734710990) [*Padr*](http://www.ine.es/dyngs/INEbase/es/operacion.htm?c=Estadistica_C&cid=1254736177011&menu=resultados&idp=1254734710990)ó[*n*](http://www.ine.es/dyngs/INEbase/es/operacion.htm?c=Estadistica_C&cid=1254736177011&menu=resultados&idp=1254734710990) ) and the MNP ([Information MNP](https://www.ine.es/prensa/mnp_prensa.htm))[.](https://www.ine.es/prensa/mnp_prensa.htm)
